# Supplementary material for: Improving semi-arid agroecosystem services with cover crop mixes
Source: PLoS One. 2024 Aug 1;19(8):e0306567. doi: 10.1371/journal.pone.0306567 (PMC11293741; doi:10.1371/journal.pone.0306567)
Supplement: Attachment — Submitted filename: Response to reviewers Improving Semi-Arid Agroecosystem.docx [file pone.0306567.s001.docx]

**Response to Reviewers**

Manuscript **PONE-D-23-38654**

**Improving semi-arid agroecosystem services with cover crop mixes**

Elizabeth A. Moore,^1^ Urszula Norton,^1&2^

**Authors would like to thank anonymous reviewers for their thoughtful comments and suggestions. Please see below how the comments were resolved.**

**Reviewer 1 Technical Comments:**

With reviewer 1, it seemed like the line numbers given in comments were not consistent with original document, so comments were addressed to the best of my ability. Reviewer 2 comments were consistent with original document.

Clarification on Cover Crop Treatments (Lines 55-59):
In the Materials and Methods section, please describe more precisely the composition and purpose of each cover crop treatment (PH, SB, NF, etc.). By comparing the specific characteristics of each cover crop treatment, the reader will be able to better understand the differences between them.

Addressed, added additional information in introduction and M&M for each cover crop treatment and beneficial characteristics of plants used in mixes.

Cover Crop Treatment Effects (Lines 65-68):
Provide an overview of the specific soil health and nutrient competition effects of each cover crop treatment. Analyzing and interpreting the results in more detail will lead to more accurate interpretations.

Addressed

Temporal Changes in Soil Parameters (Lines 71-78):
The duration of the nine-week study should be discussed in relation to any changes in soil parameters observed over this period. Various factors may be responsible for this, including variations in soil moisture or nutrient levels.

Addressed

Soil Properties Table (Lines 92-93):
Prepare a short summary of the key soil properties discussed in the manuscript (pH, EC, isotopic signature, etc.). It will be helpful for readers to have a quick reference to this table and will enhance clarity.

Addressed, all requested information is found in Table 1 and discussed in lines 128-145.

Clarity on Inorganic Fertilizer Treatment (Lines 121-128):
Explain why inorganic fertilizers were chosen and the impact they were expected to have on soil fertility. Inorganic Fertilizer Treatment Modifications should be understood by the reader from this information.

Addressed, additional information was provided in M&M (lines 175-186).

Data Presentation (Lines 175-184):
When presenting key findings, consider using visual aids like graphs or charts in conjunction with textual descriptions. Providing visual representations of complex data can make it easier for people to understand and access.

Experimental layout is included in separate tif file.

Clarify the isotope analysis methodology (Lines 228-236):
This is especially apparent in the calculation of the Biological Nitrogen Fixation (BNF) in
equations (lines 228-229, 231-232). In order to facilitate better understanding, please
provide a brief explanation or reference.

Addressed, additional information added to introduction and M&M.

Statistical Analyses (Lines 247-251):
Describe more in detail how the statistical analyses were performed in R. Make sure you
Include a description of the specific tests conducted. Please provide the details on the data
that was used for each analysis, as well as any transformations that have been applied.
Moreover, As a result, statistical approaches will be more transparent due to the availability
of information.

Addressed in M&M.

Weed Biomass Regression Analysis (Lines 319-330):
Explain how weed biomass was determined by SB biomass (soil building mix) and NF biomass (nitrogen fixation mix). Provide an explanation of the significance of this analysis in relation to weed suppression. Addressed in M&M and in discussion.

Carbon Sequestration (Lines 428-432):
Discuss the inorganic fertilizer benefit of the cover crop treatments in regard to carbon sequestration. In regard to soil health and carbon capture, discuss the implications of this finding.

Carbon sequestration term was removed and replaced with increase of soil organic carbon. This was addressed in discussions section.

Conclusion Section (Lines 440-449):
It would be beneficial to provide more information regarding the superior performance of Soil Building Mix (SB) in comparison to other cover crop mixes. The potential implications of SB's performance for soil improvement and the reasons why SB outperformed others are discussed.

Addressed in conclusions section.


Figures:

Fig 2 (Lines 308-310):
Adding more descriptive labels to the x-axis and y-axis may improve the clarity of the chart. A clear explanation of each treatment abbreviation (CON, PH, SB, NF, MM) should appear in the legend.

Addressed except for legend. An explanation for CC treatment abbreviations is in the figure description.

Table 7 (Lines 337-339):
To improve the readability of the table, the formatting should be improved. Increase the clarity of the information conveyed in each column by adding more informative column headers.

Addressed, Table 7 was removed due to duplicate information presented in Fig. 4

**Reviewer 2 Technical Comments**

I found the statistical data presentation is problematic. There are indications (for e.g., Fig 3) that the two main factors (cover crop and fertility treatments) may have significant interactions. However, the data analysis did not include investigating the interacting effects. The statistical analysis section mentions two-way ANOVA, but data presentation does not reflect that.

Tests for interactions were conducted on cover crop treatment and soil amendment and no interactions were found.

For example, Fig 2 seems to be averaged across fertility treatments which can only be done if the main effect of fertility and interaction are not significant.

Tests for interactions were conducted on cover crop treatment and soil amendment were conducted and no interactions were found.

Similarly, it is important to show the interaction between cover crop and fertility treatments in Fig 3.

No interactions were found.

The discussion section is weak, sometimes very generic, and mostly focused on BNF.

Addressed, additional discussion was provided.

Additionally, I am not sure of using the term “soil C sequestration” for slight increase in soil C for some of the treatments during this 9-week experimental period. This could be a transient increase in active soil C fractions from cover crop rhizodeposits.

Valid comment, addressed, verbiage changed to increase in soil organic carbon rather than sequestration.

L19-20: Presenting fertilizer rate as N-P-K is more typical than mass of formulation. Also applied to L123-124.

Addressed

L28: Define “IF”.

Addressed

L65-68: Complex and unclear. Consider rewriting.

Addressed

L81: Express it as SI unit

Addressed

L106: Experimental set up: It seems like compost and inorganic fertilizer were added before cover crop planting. Such large amount of N and P application during cover crop phase is a concern from nutrient management aspect. Is this the typical practice in NHP wheat rotations with fallow or cover crop? Or fertilizer is generally applied to wheat?

Addressed

L148: soil water adjustment to 7% was by mass or volume?

By mass, addressed

L224: you mean legumes absent?

Yes, good catch, addressed

L302-307: Please check the figure reference. It should be Fig 3.

Good catch, addressed

L378-382: This is fundamental knowledge and can be deleted.

Addressed

Table 7 is not completely visible.

This table has been deleted due to duplicate information being presented in Fig. 4
